# Effect of intraoperative hyperthermic intrathoracic chemotherapy after pleurectomy decortication for treatment of malignant pleural mesothelioma: a comparative study

**DOI:** 10.1007/s13304-024-01986-1

**Published:** 2024-09-21

**Authors:** Hany Hasan Elsayed, Hazem Youssef Sharkawy, Mohammed Attia Ahmed, Mohammed Abdel-Gayed, Mostafa Eldewer

**Affiliations:** 1 Thoracic Surgery Department, Faculty of Medicine, Ain Shams University, Cairo, Egypt; 2 Cardiothoracic Surgery Department, Faculty of Medicine, Ain Shams University, Cairo, Egypt

**Keywords:** Mesothelioma, HITOC, Chemotherapy, Survival

## Abstract

Malignant pleural mesothelioma (MPM) is an aggressive malignancy with few long-term survivors. Despite the dismal prognosis, hyperthermic intrathoracic chemoperfusion (HITOC) was shown to improve survival in a selective group of patients. We analyzed the influence of HITOC following pleurectomy and decortication on postoperative morbidity and overall survival for patients suffering from localized mesothelioma. From March 2017 until August 2023, 55 patients with localized pleural mesothelioma underwent pleurectomy and decortication. Thirty patients performed only surgery while 25 consecutive patients had surgery followed by HITOC with cisplatin (125 mg/m^2^) infused for 70 min at a temp of 40–43 °C. We analyzed postoperative morbidity, HITOC-related complications, and the influence of HITOC on survival. The trial was registered on 19/08/2022 as NCT05508555. The HITOC group had a mean age of 53.1 ± 8.2 years while the surgery group (non-HITOC) had a mean age of 52.1 ± 8.6 years. The HITOC group had 17 (68%) men, whereas the surgery group included 18 (60%) males. The 30-day mortality in the HITOC group was 0% vs 1 case (3.3%) in the surgery group. HITOC-related transient complications occurred in 4/25 (16%) of the HITOC group (atrial fibrillation, renal impairment and transient hypotension). Progression-free survival in the HITOC group was 8 months (95% CI 4.3–11.6) vs 6 months (95% CI 2.5–9.9) in the surgery-only group (*p* = 0.79). The overall survival time in the HITOC group was 28 months (95% CI 21.5–34.5) vs 22 months (95% CI 17.5–26.5) in the surgery-only group (*p* = 0.75). Risk factors analysis for recurrence in the HITOC group confirmed a significant role for early stages (*p* = 0.03). HITOC following pleurectomy and decortication is a safe therapeutic option that may improve survival for selected patients with localized epithelial pleural mesothelioma. Patients with earlier-stage mesothelioma are more likely to benefit from radical surgery and HITOC.

## Introduction

### Background

Malignant pleural mesothelioma (MPM) is an aggressive cancer of the pleural surface. It is associated with previous asbestos exposure, with a latency period of ∼ 40 years between fiber exposure and disease presentation [1]*.*

The link between asbestos and MPM was first noted in asbestos miners in the 1940s. Though the vast majority of patients diagnosed with MPM have a documented link to asbestos exposure, other less common etiologies include high-dose radiation exposure as well as simian virus infection [2]*.*

There are three major histologic subtypes of MPM: epithelioid, sarcomatoid, and mixed-type (biphasic). The epithelioid subtype is associated with the best outcomes, whereas the sarcomatoid subtype typically has a poor prognosis [3]*.*

Pulmonary symptoms (e.g., chest pain, dyspnea, cough) with unilateral large-volume pleural effusion in a patient with a history of asbestos exposure should raise the suspicion of MPM; however, pleural fluid cytology from thoracentesis is often nondiagnostic, even after repeated attempts. More invasive procedures, such as core needle biopsy or video-assisted thoracic surgery, have higher diagnostic yields and are frequently needed [4]*.*

Recurrence after surgical treatment alone is common and, thus, multimodality treatment including surgery is the mainstay of curative intent treatment [5]*.* The aim of radical surgery is to achieve macroscopic complete resection (MCR) [6]*.*

The concept of intracavitary delivery of chemotherapy allows exposure of the poorly vascularized tumoral tissue to high concentrations of cytotoxic agents. The treatment was first developed for peritoneal malignancy where the blood-peritoneal barrier limits passage of high doses of toxic agents into the plasma and reduces the risk of systemic toxicity. The first use of HIPEC in humans was described by in 1980 to treat a patient with pseudomyxoma peritonei. It was largely developed in Japan during the 1980s, along with techniques of cytoreduction for the treatment of peritoneal cancer originating from gastric cancer [7]*.* After almost a decade, Rush et al. [8] described the first case of hyperthermic intrathoracic chemotherapy for a patient with mesothelioma. Again, the pleural blood barrier is protective against high systemic absorption. The concept has developed over the last three decades and is now an attractive option for patients with primary and secondary pleural malignancy.

Because of late-occurring symptoms and diagnosis at an advanced stage, only few patients are eligible for a multimodal treatment approach*.* Moreover, due to the laminar tumor growth within the entire pleura, surgery alone is not able to achieve microscopic complete (R0) resection. Therefore, combined treatment modalities have been established in many centres during the last years to achieve better local tumor control with increasing overall survival [9]*.*

Surgery-based multimodality therapies have been clinically explored in the past decades. In this regard, hyperthermic intrathoracic or intrapleural chemotherapy has been used as one of the multimodality therapies. Intrapleural injection of cytotoxic drugs with hyperthermic perfusion has been proven to enhance the cytotoxic effect on tumor cells with limited systemic side effects. Potential mechanisms of hyperthermic intra-pleural or intraperitoneal chemotherapy are not only the tumor cells are directly exposed to higher concentrations of chemotherapeutic agents, but also up to 44 °C for 1-h hyperthermic exposure render the cancer cells become more sensitive to the chemotherapeutic drugs while the normal tissues are unharmed [10]*.*

In this study, we aim to compare pleurectomy decortication (P/D) alone vs combined P/D with hyperthermic intrathoracic chemotherapy (HITOC) in a tertiary centre for radical surgery for the treatment of malignant pleural mesothelioma.

## Methods

### Trial design

We conducted a retrospective prospective comparative clinical trial for patients who were referred to a tertiary thoracic surgery centre for surgical management of MPM. This clinical trial was listed on 19/08/2022 on ClinicalTrials.gov as NCT05508555. The Ain Shams University institutional review board authorized the study and Ethics approval was provided on 1/8/2022 by the IRB number: FWA 000017585. All methods were performed in accordance with the relevant guidelines and regulations. Informed consent was obtained from all subjects and/or their legal guardian(s).

### Participants

The current study selected 55 patients, divided into two groups, the HITOC group with 25 cases and the non-HITOC group with 30 cases.

The criteria for assigning participants to one group depends on the time of surgery performed. We started performing routine HITOC after radical surgery for mesothelioma in October 2020 and this group of patients were prospectively studied and compared to our historical group of patients who received only surgery from March 2017 till September 2020.

Staging of patients with malignant pleural mesothelioma who were candidates for radical surgery was based on CT chest, Positron emission tomography PET and a diagnostic thoracoscopy performed preoperatively to visualize the extent of disease and obtain sufficient tissue to appropriately diagnose histological subtype.

All patients in both groups are discussed in a multi-disciplinary team meeting MDT. Patients with good performance status and early-stage disease with non-pure sarcomatous pathology are offered the radical surgery option. The decision to administer neoadjuvant or adjuvant chemotherapy is based on disease volume. Patients with localized disease and low-volume pleural disease (< 15 mm) receive upfront radical surgery followed by adjuvant chemotherapy while patients with higher-volume disease receive neoadjuvant chemotherapy followed by radical surgery. Chemotherapy is pemetrexed-based with an aim to administer at least 4 cycles (for neoadjuvant and adjuvant protocols). Our protocol is to offer surgery four to six weeks after neoadjuvant chemotherapy. Patients receiving adjuvant chemotherapy are aimed to receive treatment six to eight weeks after surgery (with or without HITOC).

The HITOC group received cytoreductive surgery in the form of P/D + injection of intrathoracic chemotherapy (HITOC), while the other group received only P/D. All patients provided informed written consent for their surgical intervention. Inclusion and exclusion criteria are shown in Table 1.

**Table 1:** Inclusion criteria and Exclusion criteria

Inclusion criteria	Exclusion criteria
All age groups were included	Patients with IV disease
Patients with localized MPM stage I, II and low volume stage III disease	Sarcomatoid histological subtype
P/D simple or extended P/D is the main surgical procedure in all cases	Secondary pleural malignancies
The surgical approach is posterolateral thoracotomy in all cases	Patients underwent EPP instead of P/D
Epithelioid and some biphasic cases are the main histological subtypes	

### Interventions

Patients were operated on under general anaesthesia with a double-lumen endotracheal tube and were placed in the lateral decubitus position. Posterolateral thoracotomy incision was performed, then pleurectomy decortication was done either simple P/D or extended P/D in which the diaphragm and pericardium were resected as well. Before closing the chest, two chest tubes were inserted at the level of the diaphragm and directed towards the apex of the lung. Competent hemostasis is performed, and the chest is closed in layers.

In the HITHOC group: after chest closure, a heat probe is inserted in the chest from the chest tube incision. The apical chest tube is used to infuse the drug. A heated chemotherapeutic agent is circulated by a hyperthermia pump. The ipsilateral lung is maintained at a partially collapsed state during this infusion. A single chemotherapeutic agent was used; cisplatin (125 mg/m^2^); infused for 70 mins at a temp of 40–43 °C. Suction is performed through the basal chest drain. The carrier solution is peritoneal dialysis fluid 1.5% concentration. In the end, we evacuated all the perfusion solution, remove temperature probes and chest drains are connected to underwater seal.

### Outcomes

All the patients’ demographics, pre and post-chemotherapy, histological subtype, clinical staging, ICU and hospital stay days, postoperative complications, thirty-day mortality, progression-free survival, disease recurrence and overall survival were collected and recorded.

### Sample size

Sample size was calculated using PASS 11.0, where a minimum of 40 patients 20 in each group are required to achieve 80.3% power, provided that no subjects drop out in both groups. In our study, we gathered 55 cases, one group with 25 subjects and the other one with 30 subjects.

### Randomization

We followed a non-randomized convenience technique, where subjects are divided into two groups, the HITOC group with 25 cases collected prospectively between October 2020 and August 2023 and non HITOC group with 30 cases allocated retrospectively in the period between March 2017 and September 2020.

### Statistical methods

Data were collected, revised, coded and entered into the Statistical Package for Social Science (IBM SPSS) version 23. The quantitative data were presented as mean and standard deviation. Also, qualitative variables were presented as number and percentages.

The comparison between groups regarding qualitative data was done using the Chi-square test. The comparison between two independent groups with quantitative data and parametric distribution was done by using an independent *t*-test.

The confidence interval was set to 95% and the margin of error accepted was set to 5%. So, the *P* value was considered significant as the following:

*P* value > 0.05: non significant (NS).

*P* value < 0.05: significant (S).

*P* value < 0.01: highly significant (HS).

Kaplan Meier curves and log rank testing were used to study the correlation between the two interventions regarding progression-free survival and overall survival analysis.

## Results

### Baseline data

Baseline characteristics are shown in Table 2. The HITOC group had a mean age of 53.1 ± 8.2 years while the surgery group (non-HITOC) had a mean age of 52.1 ± 8.6 years. The HITOC group had 17 (68%) men, whereas the surgery group included 18 (60%) males.

**Table 2:** Baseline data

	HITOC group (*n* = 25)	Surgery group (*n* = 30)	*P* value
Age (years): mean (SD)	53.1 (8.2)	52.1 (8.6)	0.65
Sex			
Male	17 (68.0%)	18 (60%)	0.54
Pathological staging			
Epithelioid	21 (84.0%)	26 (86.7%)	0.78
Biphasic	4 (16%)	4 (13.3%)	
Staging according to TNM classification			
1a	2 (8.0%)	1 (3.3%)	0.45
1b	3 (12%)	7 (23.3%)	
II	14 (56%)	12 (40%)	
III	6 (24%)	10 (33.3%)	
Type of preop. chemotherapy			
No chemotherapy	9 (36.0%)	3 (10.0%)	0.06
Pemetrexed	10 (40.0%)	17 (56.7%)	
Pemetrexed + cisplatin	6 (24.0%)	7 (23.3%)	
Pemetrexed + carboplatin	0 (0%)	3 (10.0%)	
No. of preop. chemotherapy cycles Mean (SD)	3.48 (2.80)	4.36 (1.95)	0.19
Operation			
Simple P/D	11 (44.0%)	17 (56.7%)	0.05
Extended P/D	14 (56%)	13 (43.3%)	
ICU stay (days): mean (SD)	2.28 (0.89)	2.46 (0.57)	0.37
Hospital stay (days): mean (SD)	7.04 (3.49)	8.70 (1.70)	0.04
Postoperative prolonged air leak (defined as more than 5 days)			
No air leak	23 (92.0%)	26 (86.6%)	0.39
Prolonged air leak	2 (8.0%)	4 (13.3%)	
Other peri-op. complications			
No	20 (80.0%)	30 (100.0%)	0.25
Atrial fibrillation	1 (4.0%)	0 (0.0%)	
Kidney dysfunction	1 (4%)	0 (0.0%)	
Hypotension	1 (4%)	0 (0.0%)	
Bleeding	1 (4%)	0 (0.0%)	
Left arm weakness + lid ptosis	1 (4%)	0 (0.0%)	
Postop. chemotherapy			
No chemotherapy	9 (36.0%)	13 (43.3%)	0.06
Pemetrexed	7 (28.0%)	17 (56.7%)	
Pemetrexed + cisplatin	4 (16.0%)	0 (0.0%)	
Pemetrexed + carboplatin	1 (4%)	0 (0.0%)	
Gemzar + carboplatin	2 (8%)	0 (0.0%)	
Gemzar	1 (4%)	0 (0.0%)	
Cisplatin	1 (4%)	0 (0.0%)	

In the HITOC group, 9 (36%) cases received upfront surgery while 3 (10%) cases in the surgery group received upfront surgery.

Sixteen cases in the HITOC group received neoadjuvant chemotherapy: 10 (40%) received Pemetrexed only treatment and 6 (24%) received Pemetrexed + cisplatin treatment, while in the surgery group 27 cases received neoadjuvant chemotherapy: 17 (56.7%) received Pemetrexed only, 7 (23.3%) received Pemetrexed + cisplatin and 3 (10%) received Pemetrexed +carboplatin. The number of preoperative chemotherapy cycles was 3.48 (SD 2.80) in the HITOC group compared to 4.36 (SD 1.95) in the surgery group.

In the HITOC group, the main histopathological subtype was pure epithelioid in 21 (84%) vs 26 (86.7%) in the surgery group. Biphasic subtype was 4 (16%) in the HITOC group vs 4(13%) in the surgery group.

The clinical staging in the HITOC group was as follows: stage Ia = 2(8%), stage Ib = 3(12%), stage II = 14(56%) and stage III = 6(24%). While in the surgery group staging was as follows:

Stage Ia = 1(3.3%), stage Ib = 7 (23.3%), stage II = 12(40%) and stage III = 10(33.3%). Stage II was the most common staging operated upon in both groups.

In the HITOC group, a simple P/D was done in 11 cases (44%) compared to 17 (56.7%) cases in the surgery-only group.

The ICU stay days in the HITOC group was 2.28 (SD 0.89) days vs 2.46 (SD 0.57) days in the surgery group. The hospital stay days in the HITOC group was 7.04 (SD 3.49) days vs 8.70 (SD 1.70) days in the surgery group with a significant *P* value (*P* value=0.04).

Postoperative Complications not related to HITOC were as follows: persistent air leak lasting for more than 5 days occurred in 2 cases (8%) in the HITOC group vs 4 cases (13.3%) in the surgery group. One case was explored for bleeding in the HITOC group vs 0 (0%) in the surgery-only group. HITOC-related complications were as follows: one case developed atrial fibrillation (4%), one case (4%) had mild grade I nephropathy, one case (4%) had transient hypotension during chemo-infusion intraoperatively and one case (4%) developed left arm weakness and lid ptosis that resolved after 5 days from the onset of surgery and HITOC. Overall HITOC-related complications were 4/25 (= 16%).

At baseline, there was no significant *P* value (*P* value>0.05) regarding age, sex, preoperative chemotherapy, ICU stay days, histological subtype, clinical staging, and postoperative complications*.* However, a significant *P* value (*P* value<0.05) was detected between the two groups regarding hospital stay.

### Recurrence and survival analysis

The 30-day mortality in the HITOC group was 0% vs 1 case (3.3%) in the surgery group (*P* = 0.5) (Table 3). Progression-free survival in the HITOC group was 8 months (95% CI 4.3–11.6) vs 6 months (95% CI 2.5–9.9) in the surgery-only group (*P* = 0.79). The overall survival time in the HITOC group was 28 months (95% CI 21.5-34.5) vs 22 months (95% CI 17.5–26.5) in the surgery-only group (*P* = 0.75).

**Table 3:** Recurrence and survival analysis

	Group		*P* value
	HITOC (*n* = 25)	Surgery (*n* = 30)	
Progression free survival: median (95%CI)	8 months (95% CI 4.3–11.6)	6 months (95% CI 2.5–9.9)	0.79
Overall survival time: median (95%CI)	28 months (95% CI 21.5–34.5)	22 months (95% CI 17.5–26.5)	0.75
30 day mortality	0 0.0%	1 3.3%	0.50

#### Kaplan–Meier for recurrence

**Fig. 1:**
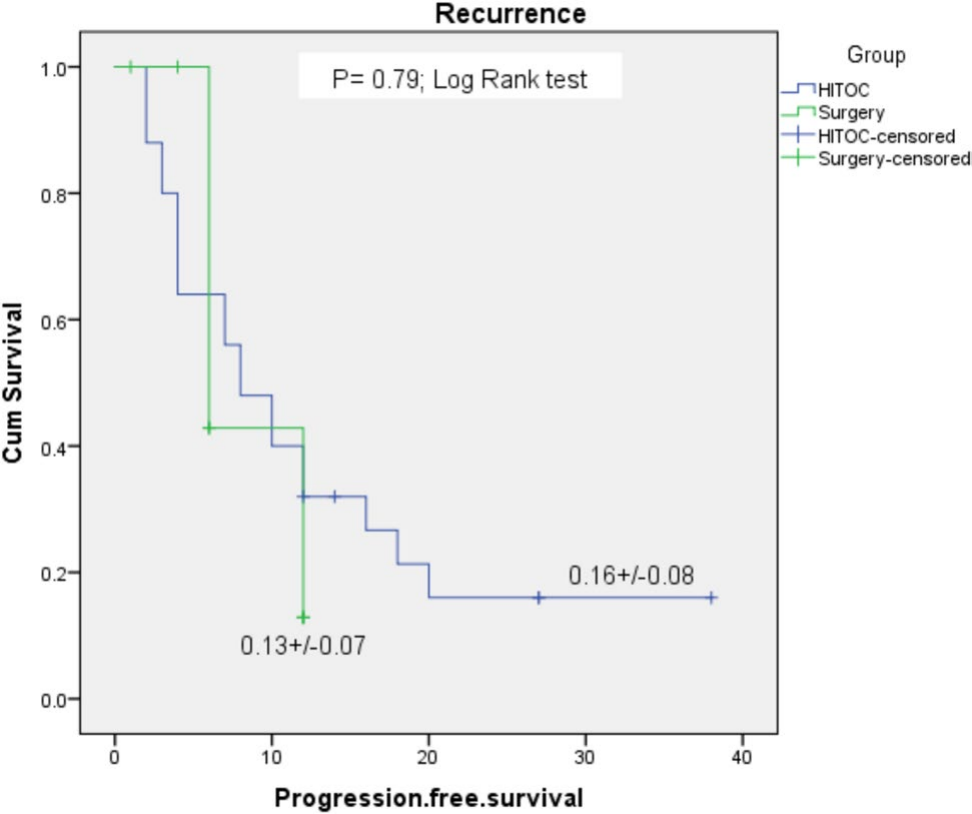
Kaplan Meier curve for recurrence

A Kaplan–Meier log-rank test was run to determine if there were differences in the progression-free survival distribution for the two groups: HITOC vs Surgery alone (Fig. 1). The progression-free survival distributions for the two interventions were statistically insignificantly different, *P* = 0.79.

#### Kaplan–Meier for survival

**Fig. 2:**
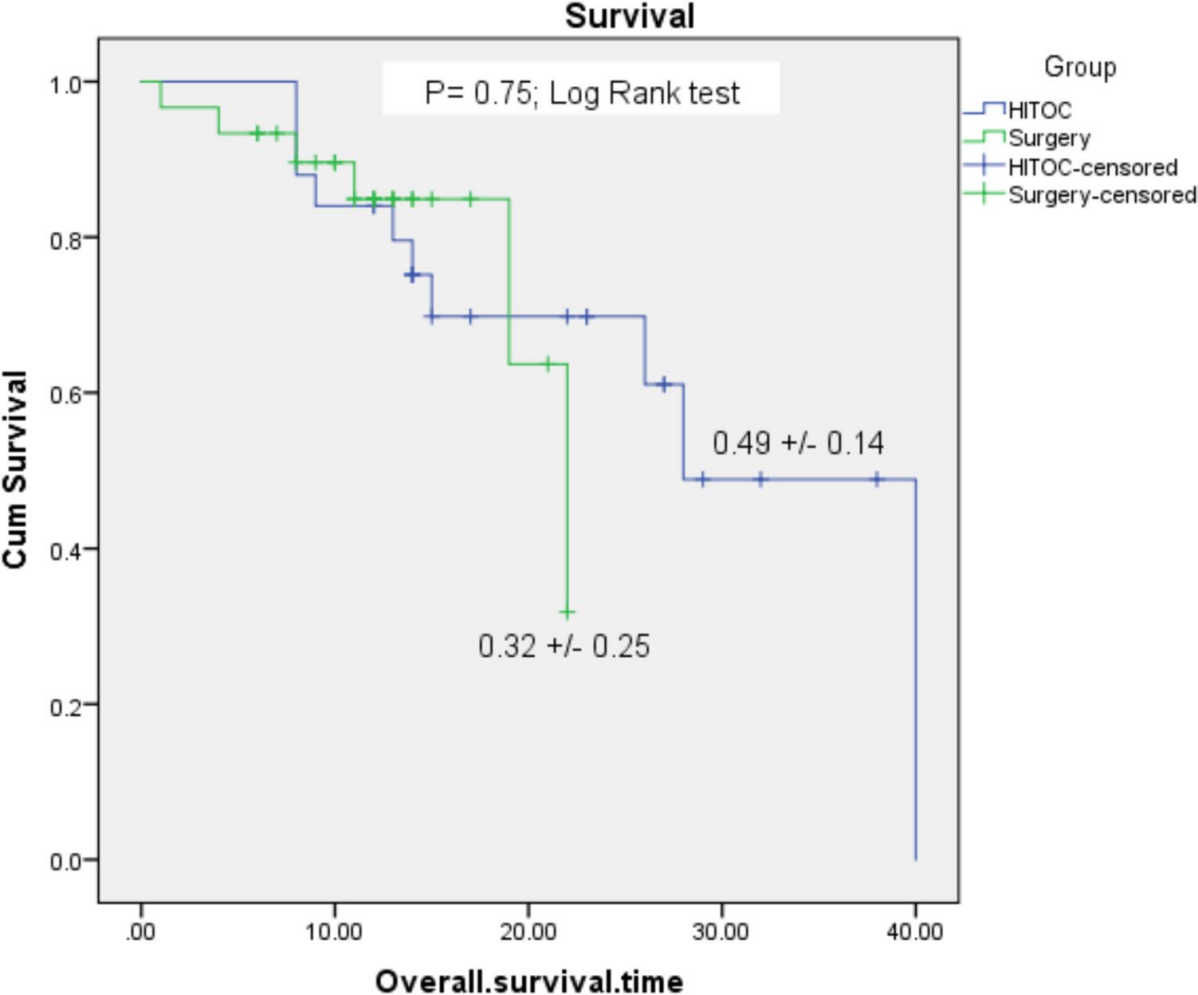
Kaplan Meier curve for survival

A Kaplan–Meier log-rank test was run to determine if there were differences in the overall survival distribution for the two groups: HITOC vs Surgery (Fig. 2). The overall survival distributions for the two interventions were statistically insignificantly different, *χ*^2^(2) = 3.39, *P* = 0.75.

#### HITOC group analysis regarding recurrence

Analysis of the HITOC group regarding recurrence as an outcome is shown in Table 4, each factor is tested independently and correlated to recurrence.

**Table 4:** HITOC group analysis regarding recurrence

	Recurrence		*P* value
	No (*n* = 13)	Yes (*n* = 12)	
Preoperative chemotherapy			
No chemotherapy	6	3	0.4
Yes	7	9	
Pathological staging			
Epithelioid	10	11	0.32
Biphasic	3	1	
Staging according to TNM classification			
1a	1	1	0.03
1b	0	3	
II	6	8	
III	6	0	
Operation			
Simple P/D	4	7	0.17
Extended P/D	9	5	
Post-operative chemotherapy			
No chemotherapy	7	2	0.11
Yes	6	10	
Number of preoperative Chemotherapy cycles			
Mean	2.62	4.41	0.11
SD	2.66	2.75	

Clinical staging of the disease is the only factor that showed a significant *P* value (*P* value = 0.03) with a correlation to recurrence.

#### HITOC group analysis regarding survival

Analysis of the HITOC group regarding survival as an outcome is shown in Table 5, each factor is tested independently and correlated to survival. No factor showed significant p-value in correlation with survival outcome.

**Table 5:** HITOC group analysis regarding survival

	Survival		*P* value
	No (*n* = 10)	Yes (*n* = 15)	
Preoperative chemotherapy			
No chemotherapy	1	8	0.35
Yes	6	10	
Pathological staging			
Epithelioid	6	15	0.88
Biphasic	1	3	
Staging according to TNM classification			
1a	1	1	0.17
1b	2	1	
II	4	10	
III	0	6	
Operation			
Simple P/D	5	6	0.18
Extended P/D	2	12	
Postoperative chemotherapy			
No chemotherapy	2	7	1.0
Yes	5	11	
Number of preoperative Chemotherapy cycles			
Mean	4.86	2.94	0.10
SD	2.27	2.86	

## Discussion

To the best of our knowledge, this is the first study to compare the results of MCR in patients with MPM utilizing HITOC with a control group of the same surgical procedure without HITOC. The advantages of using HITOC after MCR for MPM are appealing and seem to attract more surgeons in recent years to use this facility. The idea of multimodality therapy (surgery + chemotherapy + hyperthermia) in one setting fits the idea of the general need of using multi-modality in patients with mesothelioma. The low systematic absorption and hence lower morbidity and more rapid recovery are additional advantages. The procedure is usually tolerated by most patients and is compatible with all adjuvant therapies.

In a recent systematic review by Dawson et al. [11] looking at all studies utilizing HITHOC after MCR procedures, five studies [12–16] demonstrated a survival benefit for patients receiving HITOC with median survival ranging from 13 to 35 months in comparison to 11–22.8 months for the non HITOC group. Three out of the five studies [12, 14, 15] compared EPP to EPD as the surgical preference for MCR. One study [13] used povidone-iodine instead of intrathoracic chemotherapy. Only one study by Van Sandick et al. [17] found a negative outcome with HITOC in patients performing EPP (11 months vs 29 months). There was no reported mortality in relation to complications associated with HITOC.

Klotz and his colleagues [18] analyzed the clinical outcome of 71 patients affected by MPM, treated with P/D followed by HITOC with cisplatin and doxorubicin. Peri-operative morbidity appeared to be acceptable since more than 40% of all complications were classified as minor and successfully relieved by conservative treatment. They observed a median overall survival of 16.1 months, significantly influenced by histological pattern: 9.2 months for sarcomatoid, 10.9 months, for biphasic and 17.9 months for epithelioid type. Multivariate analysis confirmed that histologic tumor subtype and radicality of resection impact overall survival rates as independent risk factors.

The better survival observed in our study with HITOC patients may warrant larger studies to achieve statistical significance. Although our median overall survival in the HITOC group was not as high as in other studies, we believe that a better selection of MPM for surgery can offer better results. Patients with stage I and pure epithelioid pathology can have better survival. We also agree with Bertoglio and his colleagues [19] that diaphragmatic sparing P/D can offer better results, and this is our current surgical policy. Another study by Ambrogi et al. has also confirmed the benefit of diaphragmatic sparing surgery and shows similar results to our study of early stages of disease reducing the risk of recurrence [20]*.*

The results of this study are interestingly presented in an era questioning the benefits of surgery beforehand for mesothelioma after the results of the MARS 2 trial [21] have been recently released by Dr Lim [22]*.* OS favored chemotherapy alone for the first 42 months from randomization (hazard ratio [HR], 1.28; 95% CI, 1.02–1.60; *P* = 0.03). After 42 months, the difference in OS disappeared (HR, 0.48; 95% CI 0.18–1.29; *P* = 0.15). Progression-free survival was not significantly different between the treatment arms (HR, 0.90; 95% CI 0.72–1.11; *P* = 0.33). The risk of grade 3 or higher adverse events was greater for patients in the surgery arm than for those in the chemotherapy-alone arm (incidence rate ratio 3.6; 95% CI 2.3–5.5; *P* < 0.001). Patients in the surgery arm had a greater risk of repeat interventions; cardiac disorders; infections or infestations; and respiratory, thoracic, or mediastinal disorders. Obviously, these results are a great discouragement to offer patients radical surgery for mesothelioma, but surgeons may still see benefits to offer a selected group of patients radical surgery for MPM.

Criticism of the trial includes enrolling patients with non-epithelioid pathology with known worse prognosis and results showing that 40% of patients in the surgery arm did not receive chemotherapy cofounding the results to a comparison between surgery and chemotherapy for MPM. Also noted that there were key differences between the treatment arms at randomization when we look at the rate of diaphragmatic infiltration between the groups. Finally, nearly half (45%) of patients enrolled in MARS 2 trial were treated at centers with low surgical volumes. Patients in the surgical arm were deprived of the potential additional benefit of HITOC.

HITOC may be considered as a safe, feasible and effective local treatment to improve the local effect of surgery, but even if many studies show promising results HITOC has not been discussed in the last guidelines of the task force of the ERS/EACTS/ESTS/ESTRO on the treatment of MPM, as Migliore and colleagues have already noticed [23]*.*

We agree with Aprile and his colleagues from Italy [24] that designing a prospective randomized trial on a large series of malignant pleural mesothelioma patients represents a major challenge itself because of the rarity of the tumor and its high mortality rate. Nevertheless, to face the imminent MPM incidence peak, now more than ever it’s urgent to create a standardized protocol including HITOC, by joining all our efforts in more exhaustive, large and randomized studies.

## Conclusion

HITHOC following pleurectomy and decortication is a safe therapeutic option that may improve survival for selected patients with localized epithelial pleural mesothelioma. Patients with earlier-stage mesothelioma are more likely to benefit from radical surgery and HITOC.

## Data Availability

Data is provided within the manuscript.

## Abbreviations

HITOC: Hyperthermic intrathoracic chemotherapy
MPM: Malignant pleural mesothelioma
NCT: National clinical trial
MCR: Macroscopic complete resection
EPP: Extrapleural pneumonectomy
IRB: Institutional review board
NS: Non-significant
LN: Lymph node
ICU: Intensive care unit
EPD: Extended pleurectomy and decortication
MARS: Mesothelioma and radical surgery
OS: Overall survival
HR: Hazard ratio
CI: Confidence interval
ERS: European Respiratory Society
EACTS: European Association for Cardiothoracic Surgery
ESTS: European Society for Thoracic Surgeons
ESTRO: European Society for The Radiotherapy and Oncology
